# A Two-Stage In Silico-Guided Workflow for Forensic Toxicology: Empirical Validation via Capillary Zone Electrophoresis Prior to Mass-Spectrometric Confirmation

**DOI:** 10.3390/toxics14050451

**Published:** 2026-05-21

**Authors:** Ivan Šoša

**Affiliations:** Department of Anatomy, Faculty of Medicine, University of Rijeka, 51000 Rijeka, Croatia; ivan.sosa@uniri.hr

**Keywords:** metabolomics-guided screening, in silico metabolite prediction, capillary zone electrophoresis, electrophoretic separation, LC–MS/MS, HRMS, forensic toxicology workflow, polar and ionic analytes, analytical triage, cost-efficient toxicology

## Abstract

Medium-throughput forensic toxicology laboratories are increasingly expected to detect highly polar metabolites while working under tight resource and time constraints. To meet these requirements, a workflow is proposed that includes two stages: The first is computational metabolite prediction, followed by capillary zone electrophoresis (CZE), and the second stage is mass spectrometry (MS). The predictive step generates plausible metabolites and relevant physicochemical properties, which help guide early separation strategies. CZE then provides a rapid, low-cost way to test these predictions, identify informative samples, and exclude those unlikely to yield meaningful findings. Only samples that warrant further investigation proceed to targeted LC–MS/MS or high-resolution MS for confirmation. This approach shifts analytical effort toward the least resource-intensive stages, reducing unnecessary MS runs and improving turnaround time without compromising evidentiary standards. In practice, the workflow also improves day-to-day laboratory efficiency by overcoming equipment limitations and helping analysts focus on samples with genuine interpretive value. This stepwise combination of techniques is therefore suitable for routine forensic casework, where analytical decisions must be transparent, reproducible, and defensible.

## 1. Introduction

Forensic toxicology increasingly involves the detection of highly polar and ionic metabolites that challenge traditional chromatographic workflows [1,2]. These analytes frequently exhibit poor retention in reversed-phase LC, necessitating alternative or complementary separation strategies [3,4,5,6]. Such compounds, however, are highly amenable to electrophoretic separation techniques, where charge and size dominate their behavior in an electric field [7].

Mass spectrometry (MS) provides the definitive level of specificity required for confirmation, but its high operational cost and instrument use time create bottlenecks in medium-throughput laboratories [8,9,10,11]. High-resolution MS (HRMS) extends its reach to suspect screening and retrospective interrogation [12,13]. In medium-scale laboratories, the main limitation is often not instrument availability but the order in which techniques are applied under real workload and resource constraints [12,13,14,15]. Long lists of potential targets, heterogeneous matrices, and variable case urgency can easily saturate LC–MS/MS capacity if every sample proceeds directly to confirmation.

Against this operational background, there is a clear need for analytical strategies that prioritize early, evidence-based decision-making before committing scarce mass spectrometric resources. This work presents an analytical sequence designed to support the justification and documentation of analytical decisions. We recommend the following analytical steps:

1. In this workflow, CZE is not treated as an independent screening method but as the empirical component of the in silico analytical stage [16,17,18].
2. Only correct samples should be sent as targets to LC–MS/MS or HRMS for final confirmation [13,19,20].

Taken together, these steps reflect a deliberate shift from reflexive confirmation toward structured analytical triage. In practical terms, early low-cost analytical steps should be used to inform whether more resource-intensive confirmatory analyses are warranted [21,22,23].

Medium-throughput forensic toxicology labs currently lack a structured, evidence-based triage process for polar and ionic analytes, which leads to unnecessary use of LC–MS/MS, longer turnaround times, and increased costs [12,13,14,15,24]. Although the workflow was initially described conceptually, it is now presented as a practical, step-by-step protocol designed for such institutions. This review aims to fill this gap by presenting a metabolomics-informed in silico → CZE → MS workflow that includes well-defined decision points.

Importantly, the proposed workflow is organized into two analytical stages rather than three independent techniques. Stage I constitutes an in silico-guided empirical triage, in which computational metabolite prediction generates explicit, testable hypotheses that are experimentally evaluated using CZE [25]. Stage II comprises NS confirmation, in which LC–MS/MS or HRMS is applied only to samples that exhibit analytically meaningful behavior during Stage I.

## 2. Methodology and Conceptual Framework

To translate this rationale into laboratory practice, the conceptual workflow was formalized into a step-wise operational framework. Expanding the conceptual model into an operational framework hopefully ensures direct applicability. Detailed implementation steps for each stage of the in silico → CZE → MS sequence include parameter selection, decision points, method-adjustment rules, and validation procedures that allow laboratories to reproduce and deploy the workflow.

### 2.1. Rapid CZE That Guides In Silico Triage and Metabolite Prediction→ MS Workflow

For clarity, this workflow is organized into two analytical stages rather than three independent techniques.

Stage I constitutes an in silico-guided empirical triage stage, in which computational metabolite prediction is operationalized experimentally through CZE. In this workflow, CZE is not treated as an independent screening or confirmation method, but solely as the empirical validation arm of the in silico analytical stage [26]. CZE and in silico predictions are intentionally coupled in this workflow to form a single analytical stage. While capillary zone electrophoresis is an established and independently validated analytical methodology, its role here is specifically to provide rapid empirical testing of hypotheses generated during the in silico phase. The analytical value of this stage therefore arises from their deliberate integration rather than from either component alone.

Stage II consists of mass spectrometric confirmation, in which LC–MS/MS or HRMS is applied only to samples that demonstrate analytically meaningful behavior during Stage I.

**Figure 1:**
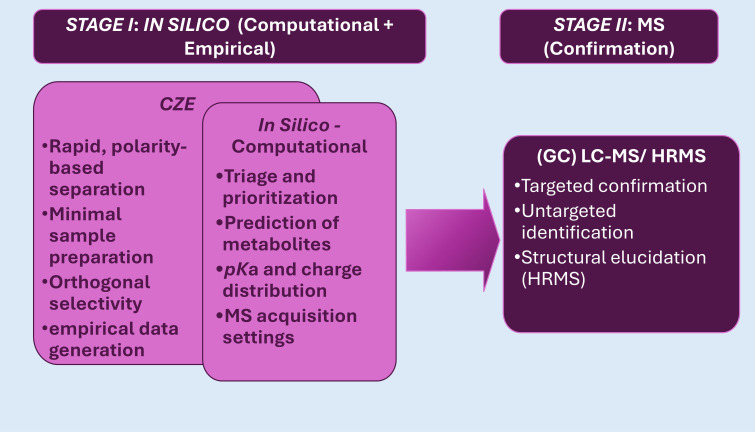
Overview of the rapid CZE that guides in silico triage and metabolite prediction → (GC)LC–MS/HRMS analytical workflow. Schematic of a two-stage forensic toxicology workflow implemented using three complementary techniques, beginning with rapid CZE that guides in silico triage and metabolite prediction and ending with targeted or untargeted MS confirmation. **Abbreviations:** CZE—capillary zone electrophoresis; GC—gas chromatography; HRMS—high-resolution mass spectrometry; LC—liquid chromatography; MS—mass spectrometry.

An overview of the proposed rapid cause-of-death exclusion (CZE) workflow is shown in Figure 1.

The workflow comprises two analytically distinct stages implemented through complementary techniques. The inception of the first stage is CZE—the first empirical checkpoint following computational triage. Here, the laboratory evaluates the predictions through empirical testing. The separations are rapid, the buffer systems are simple, and the method is highly adaptable. The result of this step/technique is an empirical profile: migration times, relative ordering, and basic selectivity information that confirm or contradict the computational expectations [18,27]. Once electrophoretic behavior has been empirically characterized, confirmatory analysis can be targeted with greater precision. CZE contributes structurally informative constraints that support the generation of candidate molecular representations—Simplified Molecular Input Line Entry System (SMILES). In this context, CZE does not generate SMILES directly; rather, it provides empirical electrophoretic descriptors—including migration order, apparent electrophoretic mobility, and pH-dependent charge behavior [28]. These mobility-derived features constrain the ionization state, formal charge, and acid–base functionality, thereby narrowing the chemically plausible space prior to structure generation. When CZE mobility patterns are integrated with accurate mass and MS/MS fragmentation data, the combined evidence can be supplied to modern computational annotation frameworks and deep learning structure generators, which output candidate molecular graphs encoded as SMILES. The orthogonal selectivity of CZE is particularly valuable for polar and ionic metabolites, where electrophoretic behavior provides information not accessible from MS alone and improves the prioritization and plausibility of generated SMILES structures [29]. This sets the stage for the next step or technique in Stage 1, a purely computational in silico step. Established data play a critical role in bridging the gap between computational prediction and empirical observation during the in silico-guided analytical stage. Initial in silico models predict metabolite structures, *pK*ₐ values, charge-state distributions, and expected electrophoretic behavior across relevant *pH* ranges [30,31]. CZE provides rapid experimental access to these same properties through observed migration times, apparent electrophoretic mobilities, migration order, and pH-dependent selectivity [32,33,34].

Comparing predicted versus observed electrophoretic behavior would enable immediate feedback to be obtained for the in silico models. Agreement between predicted charge states and measured mobilities supports the plausibility of proposed structures, whereas systematic deviations highlight incorrect ionization assumptions, missing tautomeric or protomeric forms, or unanticipated functional groups. These discrepancies can be used to iteratively refine in silico inputs—such as revising *pKₐ* estimates, constraining allowable ionization states, or pruning structurally implausible candidates—before advancing to mass spectrometric confirmation.

In this manner, CZE does not follow in silico prediction as a downstream screening step; instead, it empirically calibrates and validates the predictive space itself. The resulting in silico models are therefore no longer purely theoretical but are informed by experimental mobility data, leading to narrower suspect lists, more defensible structural hypotheses, and more efficient targeting during subsequent mass spectrometric analysis [35].

When moving on to Stage II, the third step or technique is employed—MS: targeted LC–MS/MS is used when the compounds are already well characterized, whereas HRMS is applied when the goal is discovery (non-targeted screening) or in cases of retrospective analysis.

The primary strength is that MS resources are allocated to the samples and features of greatest analytical relevance [36]. Throughout the workflow, documentation should be as disciplined as the chemistry. To situate this workflow within existing forensic and analytical practice, a structured review of the relevant literature was undertaken.

Although three techniques are employed, the workflow consists of two analytical stages. The first stage is the in silico stage, comprising computational prediction and its empirical validation using CZE. The second stage is MS confirmation.

### 2.2. Literature Review Approach

This work was conducted as a structured, concept-driven narrative review rather than a formal systematic review. Accordingly, no PRISMA or PRISMA-ScR scheme was applied. Instead, a predefined literature search strategy was used to identify representative and methodologically informative studies relevant to the integration of in silico prediction, capillary zone electrophoresis, and mass spectrometry in forensic toxicology workflows.

A focused, concept-driven review of the literature was conducted to identify the most relevant applications of integrating in silico prediction, CZE, and MS to establish a cohesive forensic toxicology workflow. The aim was to identify studies that offered clear methodological insight or practical guidance. The literature accessed spans roughly the past decade, with an emphasis on work published between 2017 and early 2026. Priority was given to research that clarified how physicochemical modeling, mobility-based separations, and MS selectivity can be arranged into a coherent analytical sequence.

Based on these sources, the proposed workflow was shaped, and the justification for placing in silico prediction and CZE as early, cost-efficient decision points before advancing samples to definitive MS confirmation was provided.

The literature review was organized around a ten-year period, capturing key methodological advances and highlighting recent studies that contributed directly to the integrated workflow. Spanning from 2017 to 2026, it aimed to capture rapid advancements in metabolomics, in silico prediction tools, CE innovations, and MS approaches, with particular emphasis on forensic toxicology.

Peer-reviewed sources were retrieved from major scientific platforms known to publish high-impact forensic and analytical research (Supplementary Table S1), including PubMed, Scopus, Web of Science, SpringerLink, Oxford Academic/JAT, Frontiers, MDPI, RSC Publishing, Wiley, and Chromatography Today. These publishers were selected because they consistently report developments in in silico metabolite prediction, CE MS methodology, and high-resolution MS confirmation, as illustrated by comprehensive reviews of metabolomics in forensic toxicology, CE MS advances in metabolomics and electrophoretic separations, and comparative evaluations of in silico prediction tools for new psychoactive substance (NPS) metabolism. Additional case-driven studies, such as HRMS confirmation workflows informed by computational metabolite prediction in fatal intoxications, were also included to ensure coverage of applied forensic scenarios. Titles, abstracts, and full texts were screened for relevance to forensic toxicology, polar/ionic analytes, CE-based separations, in silico modeling, or MS confirmation. Only English-language, peer-reviewed studies that align with the methodological themes of the present workflow were retained. This process ensured a curated evidence base reflecting real-world analytical challenges, current instrumentation trends, and the practical rationale for sequencing in silico → CE → MS within contemporary forensic toxicology. This review was guided by a focused methodological question central to contemporary forensic toxicology workflows:

How have in silico metabolite prediction tools, capillary (zone) electrophoresis (including CE–MS/CE-SDS), and mass spectrometry (LC–MS/MS, HRMS) been integrated—separately or in sequence—within forensic toxicology work to improve the triage, detection, and confirmation of polar/ionic analytes and NPS?

Rationale: This review draws upon a structured, narrative, and concept-driven approach to the literature, considering rapid advancements in metabolomics-informed forensic workflows, CE(-MS) for polar ions, and in silico metabolite prediction for NPS.

## 3. Detailed Workflow Components

Insights drawn from the reviewed literature were then integrated into a defined set of workflow components, as described below.

### 3.1. Role of In Silico Prediction in Guiding Capillary Zone Electrophoresis and Vice Versa

CZE is closely linked to in silico prediction because it tests the same properties predicted in computational analyses (charge, pH dependence, mobility). Computational tools for metabolite prediction can address different tiers of metabolomics. Predicting metabolic products involves applying a curated collection of chemical transformation rules to a substrate, similar to rule-based engines. This approach captures common biotransformations like oxidation, reduction, hydrolysis, conjugation, and ring opening. Data-driven or machine learning approaches generalize from known chemistry to propose plausible products for new scaffolds. The particular software package is not as important as the rigor with which the analyst records key details—candidate structures, predicted metabolic hotspots, and the physicochemical features that will govern how the compounds separate. In electrophoretic systems, the descriptors that provide the most insight are those that vary with *pH* [37]. A reliable estimate of *pK_a_* allows the laboratory to choose buffer conditions that place the analyte in a charge state with good mobility and minimal co-migration. Charge-state distributions can be sketched across a range of *pH* values to reveal where a family of metabolites is likely to fan out or collapse into each other. *LogD* offers a sense of how strongly matrix components might interact or how compatible a target is with subsequent LC steps. The practical result of this step is a concise plan for method development, including the candidate buffers and levels to test initially, an initial voltage and temperature settings, whether dynamic or permanent capillary coatings are needed, and, when stereochemistry matters, chiral selectors worth evaluating [38,39].

A tendency to overinterpret the precision of calculated parameters has been observed [40,41]. Such tendencies should be deliberately avoided [42]. The predictions should be employed primarily to refine the initial set of experimental conditions. When the case involves poorly characterized substances—such as emerging psychoactive compounds or biotransformation products reported in only a handful of studies—the computational step/technique is where the case accelerates. Rather than building an LC method that must be re-optimized for every tricky polar compound, the lab starts with an electrophoretic separation that is naturally tuned to charge and size. The modeling stage ensures early CZE runs are not blind.

From these general principles, practical recommendations can be derived for routine forensic implementation.

#### Recommended In Silico Software for Forensic Applications

At this foundational in silico (computational) stage of the workflow, we recommend deploying physicochemical property calculators that provide robust estimates of *pK_a*, l*ogD*, and charge-state distributions across electrophoretically relevant *pH* ranges. This specifically includes ACD/Labs PhysChem/LogD (Percepta/PhysChem Suite), MoKa (Moldiscovery), and ADMET Predictor^®^ (Simulations Plus), as the performance of these tools has been validated and they are directly applicable to predicting electrophoretic mobility and guiding CZE buffer/*pH* selection [32,33,34]. In parallel, the selection and validation of metabolite prediction engines (rule-based and/or machine learning-assisted) should follow benchmark informed practices. In this manner, it is possible to ensure defensible suspect and inclusion lists for MS confirmation; here, recent comparative evaluations of web-based toxicity and ADME prediction utilities provide practical guidance for choosing platforms that balance coverage with reliability in forensic settings [43]. In practice, the outputs from these tools (dominant ionization states, likely metabolic “hotspots,” and candidate biotransformation products) should be translated into initial CZE method parameters (buffer system, *pH* window, need for dynamic coatings) in a manner consistent with the development principles of the structured CE method [37,39], thereby tightening early triage, shortening suspect lists, and reserving confirmatory MS for analytically justified targets.

### 3.2. Capillary Zone Electrophoresis as the First Empirical Step

Without CZE, in silico predictions remain empirically untested within the workflow; without in silico guidance, CZE is applied in an exploratory rather than hypothesis driven manner. Their coupling within Stage I therefore enables rapid, hypothesis guided empirical triage.

The computational predictions gain analytical value only when tested experimentally, positioning CZE as the natural next stage in the sequence. Capillary zone electrophoresis is the first hands-on checkpoint after modeling. Its job is to turn predictions into observations quickly. The basic method is straightforward, comprising a fused-silica capillary, a conductive buffer, high voltage, and a detector. Because volumes are tiny and the system is largely aqueous, consumable costs remain low. For metabolomics-relevant targets, CZE’s strengths line up with the needs of the case. Amino acids, small organic acids, nucleotides, and inorganic ions separate cleanly when the *pH* and ionic strength are chosen to emphasize differences in mobility. When stereochemistry matters—for example, when legal status hinges on an active enantiomer—chiral selectors can be introduced to provide the additional resolution needed for interpretation [40,41].

The method development process is most effective when structured and focused on key parameters. Analysis should start with a buffer at a *pH* that places most targets in distinct charge states, as indicated by the prediction step. The migration order should be documented, and any indications of adsorption or peak tailing should be noted. If adsorption occurs, the use of a dynamic coating should be evaluated, or the ionic strength adjusted. If two features co-migrate persistently, alter the *pH* in small steps, or consider a chiral additive if the compounds are stereoisomers [44]. Each change is recorded with its impact, creating a record that explains why the final settings were selected [40,41]. CZE is not intended to replace MS but to inform whether confirmatory analysis is analytically justified. If a sample shows no meaningful ionic pattern or if predicted features are absent after reasonable adjustments, the laboratory can decide not to spend further resources. When the pattern is informative, the lab passes a targeted request to MS that includes migration times, expected charge states, and any indications of stereochemical separation.

The analytical reliability of CZE has been extensively documented outside toxicology, providing useful performance benchmarks. To contextualize the diagnostic role of CZE within forensic and biomedical applications, Table 1 summarizes representative sensitivity and specificity values reported across several established clinical and analytical CZE workflows. These examples illustrate how electrophoretic mobility-based separations can deliver high diagnostic accuracy in settings where the precise resolution of charged biomolecules is essential, providing a performance reference point for laboratories considering CZE as a screening or triage tool.

These reported performance characteristics indicate that CZE can reproducibly resolve closely related ionic species in complex matrices (as shown in Table 1).

As shown in Table 1, CZE consistently demonstrates high diagnostic accuracy across applications, with sensitivity frequently above 90% and specificity near or exceeding 98% in multiple clinically validated assays [49]. These performance characteristics demonstrate that CZE can resolve closely related ionic species with high reproducibility, even in complex biological matrices. Such characteristics translate directly to forensic toxicology, where the ability to rapidly discriminate polar and ionic compounds under low-cost, low-sample-volume conditions strengthens early-phase decision-making before MS confirmation [17].

CZE separates analytes based on their electrophoretic mobility, which depends on a molecule’s charge and size in a given buffer [7,50]. In practice, the analyst controls the pH, ionic strength, capillary dimensions, temperature, and applied voltage to tune mobility. With small injection plugs and efficient heat dissipation, separations can be fast while maintaining resolution. Because the buffers are largely aqueous and volumes are tiny, the operating costs and environmental burden are modest [26].

Performance should be evaluated in relation to the specific analytical question. For ionic and highly polar analytes, CZE can deliver well-resolved separations that reversed-phase LC struggles to achieve. Chiral resolution is accessible with the right selector. When sensitivity is limited by detector choice or matrix complexity, several avenues exist: field-amplified stacking, judicious sample cleanup, or coupling to MS.

Limitations are substantive and must be addressed transparently [40,41]. Adsorption to the capillary wall can broaden peaks and spoil reproducibility; dynamic or permanent coatings often help. Matrix effects can be significant in complex biofluids; simple cleanup steps and careful buffer selection mitigate them. Reproducibility must be demonstrated with controls and documented method parameters, especially when findings will be discussed in court [16,17].

The objective is to acknowledge the technique’s shortcomings clearly and to explain how those challenges were understood and effectively controlled. When CZE yields analytically informative patterns, its primary value lies in structuring downstream mass spectrometric confirmation.

### 3.3. Translating CZE Results Into MS Acquisition Parameters

CZE outputs are converted into MS settings through a defined sequence of technical steps.

First, all migration times are documented and mapped to expected chromatographic retention intervals using historical retention–mobility alignment or available reference standards [51]. These migration-derived windows are subsequently implemented as scheduled multiple reaction monitoring (MRM) segments for LC–MS/MS or as RT-focused inclusion list triggers for HRMS. Second, the charge state behavior inferred from CZE—based on migration order, mobility differences, and predicted *pK_a_* distributions—guides the selection of ionization polarity, expected precursor charge state, and preferred adduct forms. Third, peak morphology and co-migration patterns are used to prioritize CZE-derived features for MS interrogation: baseline-separated peaks are translated into discrete precursor targets, whereas unresolved or clustered mobility features are flagged for HRMS full-scan plus data-dependent (ddMS^2) or data-independent acquisition (DIA) to ensure comprehensive coverage [52,53]. Collision energies and fragmentation schemes are selected according to the predicted structural class of each target. This mapping ensures that CZE observations are used to directly structure the MS acquisition strategy rather than used only qualitatively [54].

#### 3.3.1. Targeted and Non-Targeted Confirmation

For targeted analysis, the inclusion list is obtained directly from the computational step, refined with what CZE actually showed. If the electrophoretic profile suggests several closely related species, the list can prioritize those masses or fragments. Where retention is weak on conventional reversed-phase LC, a short hydrophilic interaction liquid chromatography (HILIC) segment or an aqueous-compatible gradient can be introduced without derailing throughput [55,56,57,58]. For broader questions—non-targeted screening, unknowns, or discordant CZE results—HRMS offers flexibility. Molecular networking or library-assisted workflows may be used at this stage, but it is crucial to maintain a clear and well-structured narrative for effective courtroom presentation. The report should show how the CZE pattern pointed to a set of MS checks, what those checks returned, and how the combined evidence supports the finding. In routine casework, to ensure transparency and reproducibility, progression to MS must follow predefined analytical criteria.

#### 3.3.2. Criteria for Advancing Samples to Mass Spectrometry

By the time MS is utilized, the chemical space has already been narrowed through empirical methods during Stage l. Samples progress from CZE to MS only after meeting specific predefined analytical criteria:

• The electropherogram shows clear peaks that surpass the minimum signal-to-noise (S/N) ratio established by the laboratory during the validation of S/N thresholds.
• Mobility features match predicted metabolites or parent compound patterns [26].
• Peak clusters or co-migrating features indicate possible structural isomers [59].
• Unexpected mobility signals contradict in silico predictions and warrant further molecular clarification [60].
• The case involves time-critical clinical–forensic decisions where comprehensive confirmation is required [61].

Some samples may be halted for further processing with MS, only in cases when repeated CZE analysis under optimized *pH* and buffer conditions demonstrates no reproducible ionic features. This includes cases where the electropherogram results remain below validated detection limits, or the observed profile shows no analytically meaningful signals [62]. Decisions to advance or withhold are documented in the analytical record.

#### 3.3.3. Decision Rules for Selecting LC–MS/MS or HRMS

The choice between targeted LC–MS/MS and HRMS follows a transparent decision algorithm. Targeted LC–MS/MS is selected when CZE mobility patterns align with known biotransformation pathways, when the analytes appear on an established suspect or confirmation list, or when quantitative reporting is required [63]. By contrast, HRMS is deployed when CZE reveals unpredicted or atypical mobility features. In cases when the predicted metabolite space is large or poorly defined, when novel or emerging psychoactive substances are suspected, or when retrospective re-analysis may be needed [64]. HRMS is also preferred when co-migration in CZE suggests that structural isomers or isobars may require separation via accurate mass and fragmentation patterning [48]. This decision framework limits HRMS use to cases where CZE indicates unresolved or unexpected analytical features [26].

## 4. Operational Handoff Procedure: CZE → MS Confirmation

The handoff from CZE to MS follows a structured and reproducible procedure designed to translate electrophoretic observations into targeted or broad-spectrum MS acquisition settings. This ensures that informative samples progress efficiently to confirmation while minimizing the risk of overlooking analytically relevant features.

### 4.1. Validation of the Rapid CZE-Guided In Silico Triage and Metabolite Prediction → MS Handoff

To ensure reliability and prevent the loss of analytically relevant signals between techniques, the handoff undergoes routine validation. Reference compounds or metabolite standards are periodically analyzed with both CZE and MS to verify migration–retention alignment, expected charge states, and fragmentation patterns. A subset of samples with “CZE-negative” profiles is periodically subjected to HRMS full-scan analysis to confirm that no meaningful analytes were missed [62,65]. When discrepancies arise—such as a CZE peak lacking a corresponding MS signal—ionization mode, collision energies, retention time windows, or inclusion lists are adjusted and documented. All decisions, parameter mappings, and divergence analyses are recorded in the handoff log, ensuring traceability, reproducibility, and forensic defensibility.

### 4.2. Comparative Considerations Across Methods

Each analytical technique offers distinct strengths and operational advantages. GC–MS is most effective when analytes are volatile, thermally stable, or easily derivatized. LC–MS/MS handles polar and thermolabile compounds with high sensitivity and specificity. HRMS enables suspect screening and discovery and supports retrospective analysis as new information emerges. CZE contributes orthogonal selectivity and speed for ionic and highly polar targets, and it offers straightforward chiral options when regulatory or clinical interpretation depends on enantiomeric composition [40,41]. Immunoassays and other rapid screens remain useful but typically require confirmation. The integrated workflow does not exclude any of these techniques; rather, it assigns each to the stage that provides the greatest analytical advantage.

Cases that need immediate triage can start with CZE, be confirmed via LC–MS/MS, and, if needed, escalate to HRMS for more extensive analysis. [19,66]. Conversely, CZE may sometimes be omitted (e.g., existing validated LC–MS/MS methods sufficiently address the analytical objectives) [21,22,23].

#### Comparative Diagnostic Performance of Common Methods

Immunoassays offer rapid, low-cost screening but require confirmatory testing due to cross-reactivity and variable sensitivity/specificity [67,68]. GC–MS is a cornerstone for the analysis of classic volatiles and many drugs with high specificity; derivatization may be needed for highly polar or thermolabile analytes [65,67,69,70,71]. LC–MS/MS and HRMS expand coverage and enable non-targeted or suspect screening NPS and complex polypharmacy, albeit at higher costs and interpretive complexity. Confirmatory MS remains necessary for courtroom reporting [67,72,73,74,75,76,77,78].

As shown in Table 2, each method exhibits distinct sensitivity, specificity, and operational constraints [79,80].

To contextualize the complementary roles of GC–MS/LC–MS/MS, in silico prediction, and CZE within the integrated workflow, Table 3 summarizes their comparative analytical attributes. For instance, many authors [45,46,47,72,73,74,75,76,77,78,81] have reported that MS coupling can enhance CZE specificity when needed. The table highlights how each technique offers a distinct balance of sensitivity, specificity, sample preparation requirements, throughput, and cost efficiency—factors that directly determine where in the workflow each method provides the most value. This synthesis clarifies why CZE functions effectively as an intermediate, low-cost, information-rich step between computational triage and definitive MS confirmation.

As shown in Table 3, GC–MS and LC–MS/MS provide the highest levels of analytical sensitivity and specificity, but at the cost of extensive sample preparation and lower cost-efficiency for modest throughput [8,23,82]. In contrast, CZE combines moderate-to-high specificity with minimal sample preparation needs and strong cost efficiency, which makes it an effective rapid-triage tool for medium-scale forensic laboratories [19,83]. The in silico step/technique, while purely predictive, provides unmatched throughput and early-phase guidance [43].

Together, these attributes reinforce the rationale for sequencing methods so that low-cost, rapid techniques minimize unnecessary MS utilization, while high-specificity techniques are reserved for cases requiring definitive confirmation.

## 5. Discussion

The novelty of the present work lies in workflow architecture rather than in the invention or modification of analytical techniques [24,84,85]. The central claim is that in silico analysis must be empirically validated, and that CZE provides the most direct and cost-efficient experimental test of the physicochemical hypotheses generated computationally [25,86,87]. Importantly, this integration does not diminish the established standalone validity of capillary zone electrophoresis; rather, it defines its specific operational role within a decision guided forensic toxicology workflow.

Within this design, CZE is not intended to replace mass spectrometry but to prevent unnecessary MS utilization by resolving implausible hypotheses early and reserving spectrometric confirmation for chemically and empirically justified targets [17,88].

This discussion explores the practical implications of sequencing in silico prediction, CZE, and MS within a decision-guided forensic toxicology workflow. It provides examples that illustrate the broader implications of designing analytical workflows centered on decision-guided sequencing instead of merely focusing on available techniques. Instead of introducing a new analytical method, it emphasizes how organizing existing tools into a structured triage sequence can improve efficiency, interpretability, resource management, and forensic defensibility in routine cases.

Beyond conceptual advantages, successful adoption depends on attention to everyday operational details.

### 5.1. Practical Notes and Troubleshooting

Among the few practical tips to assist with daily tasks, ensuring buffers are consistently freshly prepared is essential. Clearly labeled buffers with their *pH* and date of preparation visible are of the utmost importance. Individual capillaries may vary in performance; when a capillary shows increased noise or erratic current, it should be replaced rather than troubleshoot ongoing instability [39].

A concise record should be maintained of recurrent issues—baseline drift, peak tailing, loss of resolution—and remedies that have worked should be readily available. When coupling CZE to MS, ensure careful attention is paid to the interface components and acknowledge the inherent sensitivity limitations of certain compound classes. Not every metabolite that separates beautifully will ionize well, and not every sample will yield analytically meaningful results. This is acceptable in triage. The objective is to obtain sufficient preliminary information to support an evidence-based decision regarding subsequent confirmatory analysis. Ultimately, the goal of implementing the workflow is to provide timely and well-supported analytical results/conclusions. This enables us to answer the case question with defensible speed and efficiency [88].

LC–MS/MS confirmation may be performed directly when analytically appropriate. If a case is ambiguous and polar chemistry dominates, incorporate CZE early into the analytical workflow [14,18,19].

Practical implementation steps are outlined in Table 4.

The operational impact of this sequencing is best illustrated through applied forensic examples. Recent casework examples demonstrate how the rapid CZE-guided in silico triage and metabolite prediction→ MS workflow improves analytical efficiency while maintaining evidentiary rigor. In investigations of designer benzodiazepines after death, in silico modeling refined the list of predicted metabolites to a chemically consistent group [17,19]. This enables CZE to quickly identify which candidates generated unique ionic signatures important for subsequent confirmation [88,89,90]. In the reported case series, this approach eliminated approximately 60–70% of potential MS targets in the studied datasets by excluding samples lacking analytically meaningful features, thereby reducing instrument load in those applications without compromising sensitivity [45,86]. Similarly, in emergency department (ED) settings, rapid CZE profiling has proven capable of distinguishing cationic patterns associated with substituted cathinones within minutes, enabling targeted HRMS confirmation and supporting timely clinical decision-making [91,92]. Preliminary comparative observations drawn from pilot and feasibility datasets indicate that CZE correctly identifies a high proportion (reported as approximately 80–100%) of expected highly polar or ionic metabolites suggested by in silico predictions in those limited evaluations [93,94]. At the same time, it substantially decreases unnecessary LC–MS/MS analyses.

These examples highlight the practical advantage of positioning CZE as a structured triage step: it refines suspect lists, enhances resource allocation, and improves interpretive clarity across both routine toxicology and time-critical clinical–forensic scenarios [16,17]. The overall effect is a workflow that not only complements existing chromatographic and MS-based techniques but also operationalizes metabolomics principles in a manner well suited to medium-throughput forensic laboratories [12,13,14,15,24].

#### Feasibility–Break-Even Analysis and Resource Allocation

**Figure 2:**
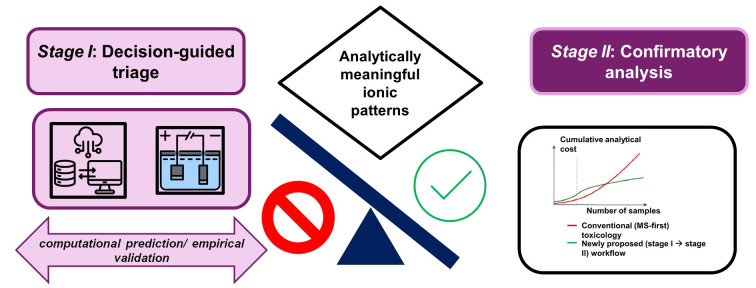
Baseline and alternative break-even cost trajectories for analytical workflows used in forensic toxicology. The baseline model reflects conventional sequencing, while the alternative model represents a decision-guided workflow in which in silico prediction and capillary zone electrophoresis (CZE) function as an upstream triage stage prior to mass spectrometric confirmation. The alternative scenario demonstrates reduced cumulative costs, primarily through decreased reliance on mass spectrometry. Values shown are illustrative and intended to demonstrate relative trends rather than absolute cost estimates.

The numeric examples shown in Figure 2 are illustrative and are intended to demonstrate relative economic trends rather than absolute laboratory costs. Computational modeling was used to explore how early decision-guided triage—implemented through the integrated in silico–CZE stage—can reduce cumulative mass spectrometric utilization and associated costs under plausible operational assumptions.

To place the proposed workflow in a practical context, it is useful to consider its economic implications through a simple break-even analysis. Budgets shape real choices. To keep the discussion grounded, consider a simple five-year amortization schedule that excludes personnel costs. In that frame, in silico workflows reach a practical break-even at roughly 200 samples, CZE at roughly 333 samples, and GC–MS at roughly 500 samples. The exact figures will vary with local pricing, maintenance plans, and consumables, but the ordering is robust: computation is cheap, electrophoresis is affordable, and confirmatory MS is the expensive step that should be reserved for the questions only it can answer. The value of CZE is most notable when considering what does not occur. Samples that do not exhibit analytically meaningful ionic features are not advanced to confirmation; suspect lists for LC–MS/MS are shorter and more focused; HRMS is saved for cases that genuinely need discovery or broad screening. These reductions in unnecessary analyses constitute the primary source of cost savings, and they are also where instrument queues shorten, and staff effort can be redirected to interpretation rather than reruns [95]. To illustrate the economic implications of sequencing analytical stages, Figure 2 compares baseline and decision-guided break-even models, highlighting how early triage reduces cumulative mass spectrometric costs.

### 5.2. Legal Admissibility and Reporting

Forensic reporting benefits from a clearly documented analytical sequence that links each technique to a defined decision point. Courts are not hostile to new combinations of familiar techniques, but they expect a clear chain of reasoning. In this workflow, the chain is short. Modeling suggests where to look and why; CZE provides a fast empirical check; MS confirms identity and, when appropriate, quantity. Each step has a clearly defined analytical role, with transitions governed by predefined and documented criteria.

Three practices are particularly influential in ensuring clarity and admissibility in court:

• First, write methods and results in plain language and keep the technical details in appendices or notes that can be produced on request.
• Second, make uncertainty visible: show ranges, not just single numbers, and explain how variability was estimated.
• Third, refrain from overclaiming. If CZE provided only triage information rather than a final identification, that should be clarified. If MS confirmation was necessary before reporting, include that detail too. Being transparent helps maintain credibility.

Court admissibility and typical forensic applications of major analytical methods are summarized in Table 5.

### 5.3. Implementation: Training, Method Development, and Validation

Training works best when it is modular and hands-on. New analysts should start with the basics of electrophoresis: how mobility depends on charge and size, how *pH* influences charge state, and how buffer composition can amplify or dampen differences. Then, instrument handling—conditioning the capillary, preparing buffers, setting voltages, monitoring current, and recognizing signs of adsorption—builds confidence [14,26,100].

Focused, well-documented trials that adjust only one parameter at a time tend to yield clearer, more reliable insight than long, complex runs [37,39]. Method development should borrow the discipline of experimental design without it becoming a burden. Select a limited set of factors that are most critical to the analysis in question—pH, ionic strength, temperature, perhaps the presence of a coating—and explore them in a structured manner [16,17]. Maintain comprehensive and well-structured documentation: buffer recipes, capillary lot numbers, voltage and temperature settings, sample preparation steps, and any issues observed. When a parameter is changed, change only that parameter and document the resulting observations. The result is a method history that explains how the final settings were chosen [17,101]. Validation must meet the requirements of the international standard for testing and calibration laboratories and the standards of the forensic community [102,103,104,105]. Linearity, limits of detection and quantification, precision, accuracy, robustness, and measurement uncertainty should be documented [106,107,108]. The laboratory should participate in proficiency testing where available, and it should keep a straightforward uncertainty budget that reflects the realities of the method rather than a theoretical ideal. None of this requires exotic statistics; it requires consistency and clarity. Standard operating procedures should make the workflow repeatable. A typical SOP will state how in silico predictions are recorded, how CZE screening is performed and interpreted, and how decisions are made about forwarding a sample to MS. It will also state how exceptions are handled and who has authority to deviate from the default path when a case demands it. Finally, the SOP should specify what is archived: raw files, processed results, and the notes that tie them together. These implementation principles translate directly into several common forensic casework scenarios.

### 5.4. Decision-Guided Toxicological Triage Workflows for Emergency Department Setting

This section does not introduce a parallel analytical pathway but rather illustrates how the proposed in silico-guided CZE → MS workflow can be adapted to time-critical forensic and emergency-driven scenarios. In such contexts, analytical value is defined less by exhaustive compound identification and more by rapid exclusion, prioritization, and escalation decisions based on physicochemical plausibility and empirical separation behavior. As such, emergency driven applications represent a practical extension of the same decision logic described earlier, operating under more restrictive temporal constraints [109,110].

Recent ED-oriented frameworks emphasize rapid triage over exhaustive early confirmation, prioritizing analytical steps that can quickly rule out major toxicological scenarios while preserving resources for cases requiring escalation [111,112]. This philosophy closely parallels the logic underpinning the proposed in silico → CZE → MS sequence, with adaptations appropriate to the clinical environment.

This approach was detailed in the recent literature from 2025 to 2026, emphasizing quick triage, organized decision-making, and transparent evidence-based processes [109,111]. Publications in merit increasingly advocate for modular, documentation-driven workflows that reduce analytical redundancy, improve traceability, and strengthen medico-legal defensibility—particularly in cases involving complex polypharmacy, new psychoactive substances, or unexplained physiological collapse.

In ED settings, the workflow begins with structured clinical and contextual assessment, often supported by algorithm-based decision tools that integrate presenting symptoms, vital signs, exposure history, and point-of-care screening results. Advances in digital triage systems and AI-assisted decision support have improved the speed and consistency with which high-risk intoxications are identified, particularly in cases involving polypharmacy, NPS, or unexplained physiological collapse [113,114].

This reliance on structured digital support extends into the early analytical phase, integrating point-of-care toxicology testing with algorithm-guided clinical decision-making. Recent progress in computational tools and multi-evidence triage systems shows that structured digital checklists and guideline-based reasoning can make early clinical assessment faster and more accurate [110,113]. Building on this shift toward a multi-tiered ED workflow, recent advances in computational triage tools further reinforce the move toward faster, structured, and more consistent early assessment [112]. Within the ED, these advances culminate in a more standardized, digitally supported workflow, as highlighted in reference [114].

**Figure 3:**
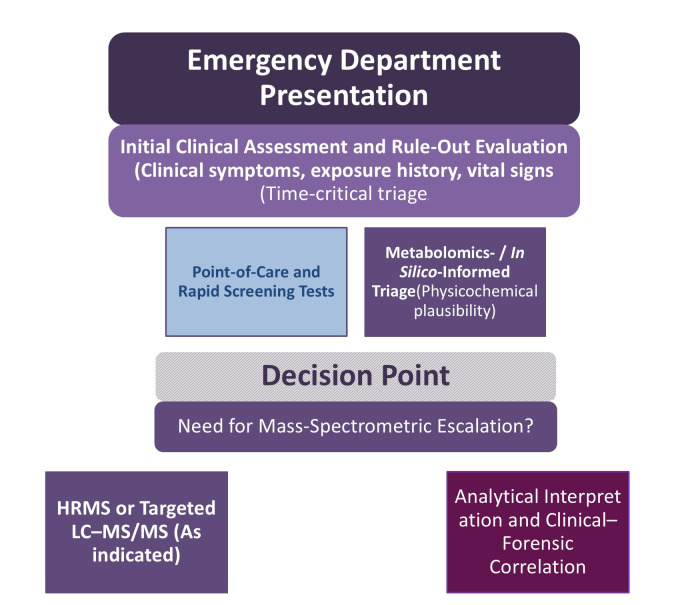
Conceptual decision logic schematic for emergency department (ED) toxicology triage integrating early rule-out assessment and mass spectrometric escalation. This schematic illustrates decision logic and information flow rather than an implemented, automated, or validated analytical system. The approach enables rapid bedside identification of toxic exposures, supports structured clinical decision-making, and improves consistency across clinical teams. Integration of metabolomics-guided triage with algorithm-supported assessment produces cleaner analytical pathways and enhances data continuity between acute care and subsequent forensic analysis. **Abbreviation**: HRMS—high-resolution mass spectrometry.

This greater dependence on structured digital tools spans from the early analytical phase, during which point-of-care toxicology testing is tightly integrated with algorithm-based clinical decision-making [115]. As shown in Figure 3, early rule-out algorithms supported by metabolomics and HRMS enable rapid bedside identification of toxic exposures and improve decision-making consistency in emergency settings. In ED contexts, escalation to mass spectrometry is often driven by clinical urgency rather than case completeness. High-resolution mass spectrometry is favored when unknown substances, atypical toxicodynamic responses, or suspected NPS exposure are involved, whereas targeted LC–MS/MS remains appropriate when suspected compounds are already defined [79,80].

Within this framework, in silico toxicological tools play a complementary role by rapidly narrowing the plausible chemical space when exposure information is incomplete. Predictive metabolite modeling and physicochemical profiling assist clinicians and laboratory personnel in anticipating classes of polar or ionic substances likely to evade conventional screening, thereby informing the selection of early analytical strategies [87,116].

Early rule-out algorithms, informed by emerging metabolomics and HRMS research, enable rapid bedside identification of toxic exposures and improve decision-making consistency across all clinical levels [117,118,119]. This forensic-aware workflow for EDs enhances clinical efficiency and produces cleaner, better-structured analytical records for forensic practitioners. This enhances the continuity between acute care interventions and later forensic analyses.

### 5.5. Typical Forensic Applications and Diagnostic Needs

Laboratories processing seized drug cases must contend with highly variable sample loads and unpredictable analytical complexity. While rapid GC–MS methods are effective for the initial screening of many common substances, samples containing highly polar analytes or metabolites often require additional clarification [120]. A short CZE run can indicate whether charged species are present, thereby signaling issues that would later interfere with LC-based separations [45,121].

A well-resolved electrophoretic profile provides immediate triage value: either proceed to MS confirmation or conclude that no analytically meaningful findings are present. Postmortem toxicology presents a different set of constraints. Matrices such as vitreous humor, bile, or tissue homogenates are chemically diverse and often contain interfering substances. CZE’s small injection volumes and tolerance for simple, aqueous buffers can be an advantage when sample is scarce or heavily processed [122]. If the case hinges on metabolic patterns rather than a single parent compound, the electrophoretic view helps structure the MS search and keeps the focus on features that matter for time-since-death estimates or pathway interpretation. Hair and other keratinized matrices require especially careful interpretive consideration [123]. Incorporation pathways, cosmetic treatment, external contamination, and pigmentation can all shift apparent concentrations or detection frequency. Although MS confirmation is essential for reporting, CZE can help in the early stages by highlighting ionic profiles consistent with true incorporation versus contamination. When the profile is inconsistent with predicted charge states or expected metabolite families, the analyst can pause and reassess the sample preparation, decontamination procedures, or overall analytical approach. Across these scenarios, the emphasis is practical. CZE does not need to answer every question; it needs to answer the first one: is there something here that justifies the next step? The same triage logic extends naturally to time-critical clinical–forensic environments.

### 5.6. Comparison with Previous Workflows and the Literature

Previous studies have explored individual elements relevant to this workflow, including in silico metabolite prediction for suspect prioritization (e.g., Bijlsma et al. [25]; Pelletier et al. [87]), capillary electrophoresis or CE–MS for polar analyte characterization (e.g., Pont et al. [17]; Schwenzer et al. [16]), and LC–MS/MS or HRMS as confirmatory or discovery tools in forensic toxicology. However, in most published workflows, these components are applied either independently or sequentially without an explicit decision-guided architecture. In particular, in silico prediction is commonly used to extend suspect lists prior to HRMS screening, rather than being empirically validated early in the analytical sequence, and electrophoretic separations are typically deployed as alternative or complementary techniques rather than as a structured triage mechanism [87].

In contrast, the present work explicitly integrates in silico prediction and CZE into a single analytical stage, where computational hypotheses are immediately tested against empirical electrophoretic behavior. Unlike CE–MS-centered approaches reported in metabolomics and proteomics-oriented studies, the objective here is not maximal molecular coverage or discovery, but rather the early exclusion of analytically implausible targets under routine forensic constraints [124,125]. This distinction is critical: while previous studies demonstrate that CE or CE–MS can resolve polar and ionic compounds with high selectivity, they generally do not formalize how this information should be used to determine whether mass spectrometric confirmation is analytically justified.

Similarly, comprehensive HRMS-based workflows described in the forensic literature emphasize broad analytical scope and retrospective capability but accept high instrument load and data complexity as inherent trade-offs [126]. The workflow proposed here differs by shifting a substantial portion of analytical decision-making upstream. Empirical observations from the reviewed case-driven literature and feasibility datasets indicate that early CZE-guided triage can eliminate a substantial fraction of predicted targets from further confirmation without compromising evidentiary rigor, thereby preserving MS capacity for samples demonstrating chemically consistent and empirically supported behavior.

Taken together, the present findings do not challenge the analytical validity of existing CE, HRMS, or in silico-centric approaches. Rather, they extend them by redefining *when* and *why* each technique is applied [35,127]. The main contribution of this work is not introducing new analytical performance data for individual techniques. Instead, it presents a decision-guided workflow architecture that harmonizes computational prediction, electrophoretic separation, and MS with the operational needs of medium-throughput forensic toxicology laboratories.

### 5.7. Limitations of the Proposed Workflow

Although the rapid CZE-guided in silico triage and metabolite prediction → MS workflow can improve analytical triage and resource allocation, several practical and methodological limitations must be considered when applying it in routine forensic work.

First, the in silico component remains fundamentally predictive [87,116]. Estimates of *pKₐ*, *logD*, charge-state distributions, and metabolic pathways are subject to model uncertainty, particularly for novel or structurally atypical compounds [128]. Prediction accuracy varies across software platforms and chemical classes, and the resulting values often reflect significant uncertainty rather than precise quantification [129]. In this workflow, in silico outputs inform initial experimental choices and should not be interpreted as confirmatory evidence of compound identity or behavior [99,130]. In practice, incorrect or incomplete predictions can lead to suboptimal initial CZE conditions or missed metabolites, underscoring the need for empirical verification in subsequent stages [131].

Second, CZE is inherently selective for ionic and highly polar analytes. Neutral, weakly ionized, or strongly hydrophobic compounds may exhibit poor electrophoretic mobility and can escape detection under typical CZE conditions. Under these circumstances, the workflow may offer limited triage benefit, and direct analysis via LC–MS/MS or alternative separation approaches may be more appropriate [132]. Furthermore, although CZE offers excellent resolution for charged species, sensitivity can be lower than that of LC–MS-based methods, depending on detector configuration, stacking efficiency, and matrix complexity [26].

Matrix effects represent an additional limitation, particularly for postmortem or heavily contaminated biological samples. Protein binding, endogenous salts, and degradation products can influence electroosmotic flow, adsorption behavior, and peak shape. While dynamic or permanent capillary coatings, buffer optimization, and minimal sample cleanup can mitigate these effects, they cannot be eliminated entirely. Rigorous validation and the use of appropriate controls are therefore essential, especially when CZE findings inform downstream confirmatory decisions [45,46,48].

Operational and institutional constraints may also limit adoption. Not all forensic laboratories maintain CZE instrumentation, and analyst familiarity with electrophoretic method development varies widely. Implementation requires targeted training, robust standard operating procedures, and disciplined documentation to ensure reproducibility and courtroom defensibility. Laboratories operating at very high throughput, or those with fully validated LC–MS/MS methods that already perform satisfactorily for the analytes of interest, may derive limited added value from introducing an additional analytical step [133].

Finally, the proposed workflow is conceptual and literature-informed and has not been validated through a single, multicenter empirical study. Although numerous published reports support individual elements of the approach, quantitative performance metrics such as false-negative rates introduced by CZE triage, long-term cost savings, and inter-laboratory reproducibility require further systematic evaluation. Future prospective studies and inter-laboratory comparisons would be valuable for defining formal performance benchmarks and refining decision thresholds [134].

Overall, these limitations highlight that the workflow should be used flexibly and with careful analysis. It is not meant to substitute established confirmatory methods but to organize early-phase decision-making transparently, defensibly, and in line with practical forensic laboratory constraints [84,85].

## 6. Future Directions

Addressing these limitations provides several clear directions for future methodological and organizational refinement. Future work should focus on strengthening the empirical and operational foundations of the proposed workflow rather than expanding its scope prematurely. One priority is the systematic evaluation of performance metrics introduced at the CZE triage step, including false-negative rates, sensitivity thresholds across different matrices, and inter-day and inter-analyst reproducibility. Multicenter or inter-laboratory studies would be particularly valuable for assessing robustness under varied instrumentation, buffer systems, and routine workload conditions.

Standardization represents another important direction. While the workflow is intentionally flexible, the development of consensus guidance for documenting in silico assumptions, CZE decision thresholds, and CZE → MS handoff criteria would improve consistency and facilitate accreditation, auditing, and courtroom communication. Template-based reporting structures and shared validation datasets could accelerate adoption without constraining analytical judgment [24,135].

Further incremental technical improvements are expected. Better coupling methods between CZE and MS, progress in electrophoretic stacking and sensitivity improvements, and the ongoing benchmarking of metabolite prediction tools are likely to increase the amount of information obtained during the initial workflow stages [47]. These developments should be evaluated pragmatically, with emphasis on measurable gains in triage efficiency rather than maximal analytical complexity [16,17].

Finally, future studies should explore how the workflow integrates with emerging digital laboratory infrastructures, including laboratory information management systems (LIMSs) and structured decision support tools. Such integration may improve the traceability and reproducibility of analytical decisions while preserving the central principle of the workflow: reserving definitive testing [136,137].

Altogether, the preceding sections support a cohesive rationale for the proposed analytical sequence.

## 7. Conclusions

The proposed workflow consists of two analytical stages. CZE functions as the empirical arm of the in silico stage, not as a standalone screening technique. The integration of in silico prediction, CZE, and MS provides a practical framework for balancing analytical cost, turnaround time, and evidentiary rigor in medium-scale forensic laboratories. The in silico analytical step narrows the problem, the electrophoretic step/technique quickly compares predictions with reality, and the MS step establishes identity with the required specificity. This arrangement does not require any technique to be applied beyond its intended use. It uses the strengths of each in a deliberate sequence and documents the handoffs so that the reasoning is visible to peers, auditors, and the court. In practice, it shortens turnaround time, reduces the number of low-value confirmatory runs, and protects instrument capacity for the questions that genuinely require it. Overall, the workflow supports the timely generation of analytically defensible results while maintaining traceable decision-making throughout the process.

## Figures and Tables

**Table 1:** Diagnostic performance of capillary zone electrophoresis (CZE) across selected clinical and analytical applications. The table summarizes representative sensitivity, specificity, and performance notes for established CZE-based assays, illustrating the technique’s reproducibility and diagnostic accuracy across diverse biomolecular targets. Values vary depending on buffer composition, capillary coatings, detector configuration, and analytical context.

Application	Sensitivity	Specificity	Notes
Monoclonal protein detection	~95%	~98.6%	Superior to AGE for low concentrations
Plasma cell neoplasm indices	95–100%	High	Validated sharpness and light-chain indexes
Haemoglobinopathy screening	>95%	>98%	Variant Hb and β-thalassemia traits
Body fluid protein classification	~90–95%	~95–98%	Reproducibility advantage over AGE

Values vary with buffer, coating, and detector [45,46,47,48].

**Table 2:** Comparative diagnostic performance of commonly used forensic toxicology analytical methods. Summary of sensitivity, specificity, analytical scope, strengths, and limitations for major instrumental and immunochemical approaches routinely employed in forensic toxicology, including GC–MS, LC–MS/MS, LC–HRMS, immunoassays, and headspace GC–FID. The table highlights how each method differs in accuracy, analyte coverage, and operational constraints, providing context for their complementary roles within integrated workflows.

Method	Sensitivity (%)	Specificity (%)	Detection Scope	Strengths	Limitations
GC–MS	85–95	95–99	Volatile/semi-volatile drugs	High specificity; robust	Limited for highly polar/thermolabile
LC–MS/MS	90–99	95–99	Broad incl. NPS	Excellent for polar/thermolabile	Complex prep/calibration
LC–HRMS	92–98	90–98	Untargeted/NPS	Detects unknowns/metabolites	High cost; complex data
Immunoassays	70–90	85–95	Targeted classes	Rapid; low cost	False positives/negatives
Headspace GC–FID	80–90	90–95	Alcohols/volatiles	Simple; reliable for ethanol	Limited scope

**Abbreviations**: GC—gas chromatography; GC–FID—gas chromatography–flame ionization detection; GC–MS—gas chromatography–mass spectrometry; HRMS—high-resolution mass spectrometry; LC—liquid chromatography; LC–MS/MS—liquid chromatography–tandem mass spectrometry; MS—mass spectrometry.

**Table 3:** Comparative analysis of GC–MS/LC–MS/MS, in silico prediction, and CZE attributes. Overview of key analytical characteristics—including sensitivity, specificity, throughput, sample preparation requirements, cost-efficiency, and validation demands—across three complementary approaches used within the integrated forensic toxicology workflow. The table highlights how each technique provides distinct strengths that support their sequential use from computational triage to electrophoretic screening and definitive mass spectrometric confirmation.

Parameter	CZE	In Silico	GC–MS/LC–MS/MS
Sensitivity	Moderate	Variable	High
Specificity	Moderate–High	Predictive only	High
Throughput	Moderate	Very High	High
Sample preparation	Minimal	None	Extensive
Cost efficiency	High (at low/medium throughput)	High	Low (at low throughput)
Validation	Moderate	Limited	Extensive

**Abbreviations**: CZE—capillary zone electrophoresis; GC—gas chromatography; GC–MS—gas chromatography–mass spectrometry; LC—liquid chromatography; LC–MS/MS—liquid chromatography–tandem mass spectrometry; MS—mass spectrometry.

**Table 4:** Practical implementation steps of rapid CZE-guided in silico triage and metabolite prediction → MS workflow.

Step	Required Actions	Outputs	Decisions
In silico	Estimate *pK_a_*/logD, predict metabolites	Charge-state map	Select CZE pH, initial buffer
CZE	Optimize *pH*, identify peaks, adjust coatings	Electropherogram	Advance vs. exclude sample
MS	Set RT windows and inclusion lists, choose modality	Confirmatory spectra	Targeted vs. HRMS workflow

**Table 5:** Court admissibility and typical forensic applications of major analytical methods. Summary of acceptance rates, common forensic uses, and key interpretive notes for CZE, GC–MS, and LC–MS/MS in legal contexts, outlining how their analytical reliability and historical validation support courtroom admissibility.

Method	Court Acceptance Rate	Typical Use in Forensics	Notes
CZE	Accepted since 1996 for DNA STR; >90% of forensic DNA labs worldwide [31,96]	DNA profiling (STR); human identification	Foundational validity for single-source and two-person mixtures [38,97]
GC–MS	>95% of toxicology and drug cases	Drug analysis; toxicology; arson	Gold standard for chemical identification; very low error rates [98]
LC–MS/MS	>90% of forensic toxicology confirmations	Drug confirmation; metabolite detection	Highly sensitive and specific; increasingly replacing GC–MS [99]

**Abbreviations:** CZE—capillary zone electrophoresis; GC—gas chromatography; GC–MS—gas chromatography–mass spectrometry; LC—liquid chromatography; LC–MS/MS—liquid chromatography–tandem mass spectrometry; MS—mass spectrometry; STR—short tandem repeat.

## Data Availability

No new data were created or analyzed in this study. Data sharing is not applicable to this article.

## Supplementary Materials

The following supporting information can be downloaded at: https://www.mdpi.com/article/10.3390/toxics14050451/s1, Supplementary Table S1 summarizes the search strategies and key publications from major scientific platforms. It includes the exact search strings used for each database or publisher (PubMed, Scopus, Web of Science, SpringerLink, Oxford Academic/JAT, Frontiers, MDPI, RSC Publishing, Wiley, and Chromatography Today), along with the specified time window (2017–2026), language filter (English), and representative publications retrieved by each search [3,18,16,87,92,138,139,140,141,142,143,141,144,145,146,89,143].

## Abbreviations

ADMET	absorption, distribution, metabolism, excretion, and toxicity
AGE	agarose gel electrophoresis
AI	artificial intelligence
CE	capillary electrophoresis
CE–MS	capillary electrophoresis–mass spectrometry
CE–SDS	capillary electrophoresis–sodium dodecyl sulfate
CZE	capillary zone electrophoresis
ddMS^2^	data dependent tandem mass spectrometry
DIA	data independent acquisition
DNA	deoxyribonucleic acid
ED	emergency department
ELISA	enzyme linked immunosorbent assay
GC	gas chromatography
GC–FID	gas chromatography–flame ionization detection
GC–MS	gas chromatography–mass spectrometry
HILIC	hydrophilic interaction liquid chromatography
HRMS	high resolution mass spectrometry
HS GC FID	headspace gas chromatography–flame ionization detection
IC HRMS	ion chromatography–high resolution mass spectrometry
ISO/IEC 17025	International Organization for Standardization/International Electrotechnical Commission laboratory standard
LC	liquid chromatography
LC–HRMS	liquid chromatography–high resolution mass spectrometry
LC–MS/MS	liquid chromatography–tandem mass spectrometry
LIMS	Laboratory Information Management System
logD	distribution coefficient
MRM	multiple reaction monitoring
MS	mass spectrometry
NPS	new psychoactive substances
pKₐ	acid dissociation constant
PRISMA	Preferred Reporting Items for Systematic Reviews and Meta Analyses
PRISMA ScR	PRISMA extension for Scoping Reviews
QA/QC	quality assurance/quality control
RP LC	reversed phase liquid chromatography
RT	retention time
S/N	signal to noise ratio
SFC	supercritical fluid chromatography
SMILES	Simplified Molecular Input Line Entry System
SOP	standard operating procedure
SNP	single nucleotide polymorphism
STR	short tandem re
