# Supplementary material for: A Two-Stage In Silico-Guided Workflow for Forensic Toxicology: Empirical Validation via Capillary Zone Electrophoresis Prior to Mass-Spectrometric Confirmation
Source: Toxics. 2026 May 21;14(5):451. doi: 10.3390/toxics14050451 (PMC13211538; doi:10.3390/toxics14050451)
Supplement: Supplementary file 1 [file toxics-14-00451-s001.zip › toxics-4279451-supplementary.pdf]

Supplementary Table ST1 Search strategies and representative literature outputs across major scientific platforms.This table summarizes the exact search strings used for each database or publisher platform (PubMed, Scopus, Web of Science, SpringerLink, Oxford Academic/JAT, Frontiers, MDPI, RSC Publishing, Wiley, Chromatography Today), together with the applied time window (2017–2026), language filter (English), and representative publications returned by each query. Representative items include, for example, studies on in-silico metabolite prediction for novel psychoactive substances, high-resolution MS confirmation workflows in forensic toxicology, forensic metabolomics investigations such as UHPLC-MS profiling in fatal hypothermia cases, and comprehensive reviews describing the role of metabolomics in forensic sciences.

**Platform**	**Search String**	**Time Window**	**Language**	**Representative Results (example titles)**
PubMed / MEDLINE	("forensic toxicology"[tiab] OR "forensic science"[tiab] OR forensic[tiab]) AND ("in silico"[tiab] OR "metabolite prediction"[tiab] OR QSAR[tiab] OR ADMET[tiab] OR BioTransformer[tiab] OR GLORYx[tiab] OR SyGMa[tiab]) AND ("capillary electrophoresis"[tiab] OR CZE[tiab] OR "CE-MS"[tiab] OR "capillary electrophoresis mass spectrometry"[tiab] OR "CE-SDS"[tiab]) AND ("mass spectrometry"[tiab] OR "LC-MS"[tiab] OR "LC-MS/MS"[tiab] OR HRMS[tiab] OR "high-resolution mass spectrometry"[tiab]) AND ("2017/01/01"[dp] : "2026/03/17"[dp]) AND (english[lang])	2017–2026	English	In Silico Forensic Toxicology: Is It Feasible? (Toxics, 2025); Applications of Metabolomics in Forensic Toxicology and Forensic Medicine (IJMS, 2021); Metabolomic analysis of fatal hypothermia using UHPLC–MS (Frontiers in Molecular Biosciences, 2025)

Scopus	TITLE-ABS-KEY ("forensic toxicology" OR "forensic science" OR forensic) AND TITLE-ABS-KEY ("in silico" OR "metabolite prediction" OR QSAR OR ADMET OR BioTransformer OR GLORYx OR SyGMa) AND TITLE-ABS-KEY ("capillary electrophoresis" OR CZE OR "CE-MS" OR "capillary electrophoresis mass spectrometry" OR "CE-SDS") AND TITLE-ABS-KEY ("mass spectrometry" OR "LC-MS" OR "LC-MS/MS" OR HRMS OR "high resolution mass spectrometry") AND PUBYEAR > 2016 AND PUBYEAR < 2027 AND (LIMIT-TO (LANGUAGE, "English"))	2017–2026	English	Identifying metabolites of new psychoactive substances using in silico prediction tools (Archives of Toxicology, 2025); In silico metabolite prediction and LC-HRMS confirmation for a nitazene fatality (JAT, 2025 online first); Capillary Electrophoresis–Mass Spectrometry for Metabolomics: Addressing Perceived Misconceptions (Chromatography Today, 2021); Profiling acidic metabolites by CE–MS using a novel derivatization approach (Analytical Science Advances, 2021)
Web of Science Core Collection	TS=(("forensic toxicology" OR "forensic science" OR forensic) AND ("in silico" OR "metabolite prediction" OR QSAR OR ADMET OR BioTransformer OR GLORYx OR SyGMa) AND ("capillary electrophoresis" OR CZE OR "CE-MS" OR "capillary electrophoresis mass spectrometry" OR "CE-SDS") AND ("mass spectrometry" OR "LC-MS" OR "LC-MS/MS" OR HRMS OR "high resolution mass spectrometry")) AND PY=(2017-2026) AND LA=(English)	2017–2026	English	Applications of Metabolomics in Forensic Toxicology and Forensic Medicine (IJMS, 2021); Metabolomic analysis of fatal hypothermia using UHPLC–MS (Frontiers in Molecular Biosciences, 2025); Forensic Narcotics Drug Analysis: State-of-the-Art Developments and Future Trends (Processes, 2025)
SpringerLink	("forensic toxicology" AND "capillary electrophoresis") AND ("mass spectrometry" OR HRMS)	2017–2026	English	Identifying metabolites of new psychoactive substances using in silico prediction tools (Archives of Toxicology, 2025); In vivo and in vitro metabolism of the designer benzodiazepine bretazenil (Archives of Toxicology, 2026 volume / 2025 online first); Recent advances and applications of CE–SDS for mAbs (The Analyst, 2026); Artifact during reducing CE–SDS analysis of a monoclonal antibody (Pharmaceutical Research, 2022)
Oxford Academic (Journal of Analytical Toxicology)	("in silico" AND metabolite) AND (forensic OR toxicology) AND (HRMS OR "high-resolution")	2017–2026	English	In silico metabolite prediction and LC-HRMS confirmation for a nitazene fatality (JAT, 2025 online first); Designer Benzodiazepines Gidazepam and Desalkygidazepam: What Do We Know? (JAT, 2023)
Frontiers (publisher)	forensic AND metabolomics AND (HRMS OR "mass spectrometry")	2017–2026	English	Metabolomic analysis of fatal hypothermia using UHPLC–MS (Frontiers in Molecular Biosciences, 2025)

https://doi.org/10.3390/xxxxx

MDPI (publisher: IJMS, Toxics, Processes)	(forensic toxicology AND metabolomics) OR ("capillary electrophoresis" AND metabolomics)	2017–2026	English	Applications of Metabolomics in Forensic Toxicology and Forensic Medicine (IJMS, 2021); Forensic Narcotics Drug Analysis: State-of-the-Art Developments and Future Trends (Processes, 2025); In Silico Forensic Toxicology: Is It Feasible? (Toxics, 2025)
RSC Publishing (The Analyst)	("capillary electrophoresis" AND SDS) AND (review OR advances) AND monoclonal	2017–2026	English	Recent advances and applications of CE–SDS for mAbs (The Analyst, 2026)
Wiley / Chemistry Europe	("capillary electrophoresis" AND metabolomics) AND (derivatization OR "acidic metabolites")	2017–2026	English	Profiling acidic metabolites by CE–MS using a novel derivatization approach (Analytical Science Advances, 2021)
Chromatography Today	("CE-MS" AND metabolomics) AND reproducibility	2017–2026	English	Capillary Electrophoresis–Mass Spectrometry for Metabolomics: Addressing Perceived Misconceptions (2021)
Analyte Class: Cathinones (any platform)	(cathinone* AND "capillary electrophoresis") AND (pKa OR acidity OR enantio*)	2017–2026	English	Acidity of substituted cathinones studied by capillary electrophoresis (Talanta, 2018); Enantioselective separation of synthetic cathinones by CE with ionic liquid and cyclodextrin co-additives (MDPI Separations, 2023)
Analyte Class: Designer Benzodiazepines / NPS (any platform)	("designer benzodiazepine*" OR DBZD* OR nitazene*) AND (forensic OR toxicology) AND (HRMS OR LC-MS/MS)	2017–2026	English	Forensic toxicology of benzodiazepines: analytical challenges and emerging detection strategies (Frontiers in Toxicology, 2025); Bretazenil metabolism using in silico, in vitro, in vivo and HRMS (Archives of Toxicology, 2026 volume / 2025 online first)

https://doi.org/10.3390/xxxxx
